# Conformational States of Macromolecular Assemblies Explored by Integrative Structure Calculation

**DOI:** 10.1016/j.str.2013.08.006

**Published:** 2013-09-03

**Authors:** Konstantinos Thalassinos, Arun Prasad Pandurangan, Min Xu, Frank Alber, Maya Topf

**Affiliations:** 1 Institute of Structural and Molecular Biology, Division of Biosciences, University College London, London WC1E 6BT, UK; 2 Institute of Structural and Molecular Biology, Birkbeck College, University of London, London WC1E 7HX, UK; 3 Molecular and Computational Biology, University of Southern California, 1050 Childs Way, Los Angeles, CA 90089, USA

## Abstract

A detailed description of macromolecular assemblies in multiple conformational states can be very valuable for understanding cellular processes. At present, structural determination of most assemblies in different biologically relevant conformations cannot be achieved by a single technique and thus requires an integrative approach that combines information from multiple sources. Different techniques require different computational methods to allow efficient and accurate data processing and analysis. Here, we summarize the latest advances and future challenges in computational methods that help the interpretation of data from two techniques—mass spectrometry and three-dimensional cryo-electron microscopy (with focus on alignment and classification of heterogeneous subtomograms from cryo-electron tomography). We evaluate how new developments in these two broad fields will lead to further integration with atomic structures to broaden our picture of the dynamic behavior of assemblies in their native environment.

## Teaser

Thalassinos et al. provide a perspective on the latest advances and future challenges in computational methods of interpreting data from MS and cryo-electron microscopy and electron tomography. They discuss developments that will lead to further integration of the techniques to broaden our understanding of assemblies in their native environment.

## Main Text

Cellular processes, from signaling to metabolic pathways, molecular transport, and gene expression, are governed by interactions of proteins and nucleic acids often forming macromolecular assemblies. Detailed structural characterization of such assemblies is essential for gaining a deeper understanding of how cells operate and how they evolved. At present, subunit compositions, topology, overall architectures, and conformational dynamics of most assemblies are unknown. It is becoming clear that the integration of data derived from a variety of biophysical techniques at multiple levels of resolution can achieve structural analysis of large assemblies that are otherwise refractory to high-resolution structure determination with X-ray crystallography or nuclear magnetic resonance (NMR) spectroscopy (Alber et al., 2008; Karaca and Bonvin, 2013; Lander et al., 2012; Ward et al., 2013). Naturally, some of these techniques have become more prominent in recent years, especially due to their complementary nature as well as their ability to deal with large heterogeneous complexes.

One such example is mass spectrometry (MS). MS measures the mass-to-charge ratio (*m/z*) of ionized species from which the mass of a particular ion can be determined. In the past two decades, MS has become a key technology in proteomics, where measuring masses of peptides and their fragments combined with detecting their identity by database searches is used to identify proteins in the sample on a molecular, cellular, or organismal level (Angel et al., 2012; Cox and Mann, 2011; Kühner et al., 2009). In parallel, MS is increasingly used for analyzing intact proteins (often referred to as native MS) and their assemblies with other proteins, DNA, RNA, and small molecules because it is possible to maintain such interactions during the transfer to the gas phase (Heck, 2008; Sharon and Robinson, 2007). It can reveal the oligomerization state of the protein, the number of ligands bound to it, and, for protein complexes, their overall composition, subunit stoichiometry, as well as its architectural organization. Additionally, insights into the conformational dynamics of the system can be obtained by combining MS analysis with sample preparation approaches such as chemical crosslinking, hydrogen-deuterium exchange, as well as other gas-phase separation approaches such as ion-mobility MS (Hyung and Ruotolo, 2012; Walzthoeni et al., 2013).

A second example is three-dimensional (3D) cryo-electron microscopy (3D EM). Because of advances in cryogenic methods, instrumentation, and image processing, 3D EM has demonstrated many biological objects in a close to native, hydrated state (Orlova and Saibil, 2011). In single-particle cryo-EM, the general assumption is that the studied objects in the sample (“particles”) are identical and therefore their images can be classified and averaged (to improve signal-to-noise ratio) into different two-dimensional (2D) “views” and subsequently reconstructed into a 3D map. In cryo-electron tomography (cryo-ET), a tomogram is reconstructed from a set of 2D cryo-electron micrographs, which are collected by tilting one sample around a single rotational axis. Therefore, this technique depicts pleomorphic biological specimens (Leis et al., 2009). Cryo-ET has revealed low-resolution ultra-structures of whole cells and the distributions of large complexes inside them (Beck et al., 2009; Yahav et al., 2011; Gan and Jensen, 2012). Increasingly, the single-particle approach is also used in cryo-ET (via subtomogram averaging) to generate higher resolution 3D density maps of individual assemblies, and therefore is becoming particularly popular in studying large assemblies, including those bound to membranes (Al-Amoudi et al., 2011; Briggs, 2013; Maurer et al., 2013).

Naturally, cryo-EM/ET methods can capture complexes during vitrification in different conformational states, which are often biologically relevant (Fourniol and Moores, 2011). In cryo-EM, statistical analysis and classification methods have been applied with great success in detecting conformational differences in assembly structures (Agirrezabala et al., 2012; Clare et al., 2012). In cryo-ET, there is great potential, as the technique explores macromolecular assemblies in their natural environment (Beck et al., 2004). However, many challenges remain, not only due to lower resolution and lower signal-to-noise ratio, but also due to distortions in the sample as a result of the missing wedge effect and the increase in computational costs when processing 3D instead of 2D images.

Indeed, progress in sample preparation, instrumentation, as well as data processing and interpretation in both 3D EM and MS has had a tremendous impact in studying the architecture of macromolecular assemblies in a complementary fashion (Ortiz et al., 2010; Lasker et al., 2012; Malet et al., 2012; Noble et al., 2013; Anger et al., 2013; Housden et al., 2013). Yet this progress has brought with it many new challenges, especially regarding the analysis of conformational states in assemblies. This Perspective aims to specifically address this latter issue. We discuss recent developments and future challenges in computational methods that aid the analysis of these techniques and the integration with atomic models to enable detailed insights into the dynamic nature of macromolecular assemblies.

### Probing the Dynamics of Assemblies with Mass Spectrometry

Computational methods are at the heart of proteomics research as the vast amount of data generated need to be processed, searched against protein databases, quantitated, efficiently stored, and linked to public pathway and interaction databases. There are numerous programs, both commercial and free, developed for such a purpose, but the type of sample preparation, experimental design, and particular MS instrument used for the analysis often dictate which programs are to be used.

**Figure 1:**
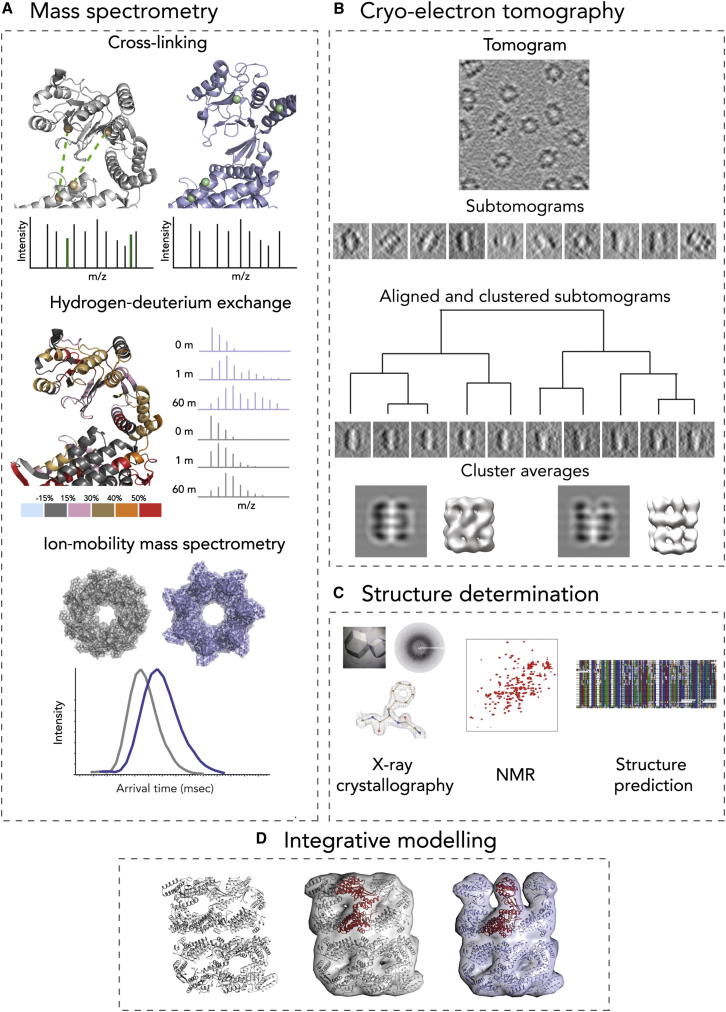
Diverse Types of Information Calculated Based on Data from Various Techniques about a Dynamic Complex (A–C) Information based on MS techniques, including peptides indicating crosslinks, solvent exposure information from HDX-MS, and arrival time distributions from IM-MS that can be used to calculate CCSs (A), 3D density maps of two conformational states detected from cryo ET classifications (B), and atomic models of the complex resulting from X-ray crystallography, NMR spectroscopy, or structure prediction methods (C). (D) The information can be combined to provide pseudo-atomic models of the complex in multiple conformations.

Chemical crosslinking coupled to MS (XL-MS) reveals not only which proteins interact in the sample, but also which specific parts are involved in the interaction (Rappsilber, 2011; Figure 1A). A challenge arises because crosslinked peptides are usually in much lower abundance compared to non-crosslinked ones; therefore, protein fractionation steps, based on strong cation exchange, are often used to circumvent this problem (Leitner et al., 2012b). The number of possible crosslinked peptide combinations, from even a small number of proteins, is very high. This problem drastically increases the search space of the database to be considered, because each peptide from a given protein needs to be in silico crosslinked with every other peptide from the other proteins in the database (Rappsilber, 2011). As a result, the false discovery rate increases. Additionally, the fragmentation of crosslinked peptides gives rise to complex tandem mass spectra (further complicating database searches). Programs have recently been developed to circumvent some of these issues (Walzthoeni et al., 2012; Yang et al., 2012).

Despite these challenges, XL-MS has been successfully used to capture the dynamics of large protein complexes because crosslinking can freeze transient protein interactions. An example is the study of ribosomal protein S1 by Lauber and colleagues, which revealed that the N-terminal part of the protein binds the ribosome while the C-terminal part is very dynamic and used as an mRNA catching arm (Lauber et al., 2012). Another recent example has used comparative crosslinking to study the dynamics of chloroplast ATPase upon phosphorylation (Schmidt et al., 2013). The study was able to shed light on how phosphorylation changes subunit interactions in the complex thereby regulating nucleotide binding.

Hydrogen-deuterium exchange coupled to MS (HDX-MS) can provide information regarding the flexibility of certain parts of a protein. For example HDX-MS experiments can reveal protein dynamic events during protein folding/unfolding, conformational changes (e.g., induced by ligand binding), hydrogen bonding, or solvent exposure. While HDX experiments are not trivial, recent developments in both automated sample preparation, MS instrumentation, and enhanced data analysis algorithms (Kazazic et al., 2010; Pascal et al., 2009; Zhang et al., 2012b) have increased the use and reliability of the technique (Iacob and Engen, 2012). Because the buried residues generally exchange hydrogens more slowly than the surface residues, HDX-MS results reflect differences in solvent exposure by comparison of the same peptide segments from free and bound proteins in a complex (Noble et al., 2013). The resolution of the approach is limited by the size and overlap of the identified peptides generated after proteolysis of the labeled proteins. In some reports, near-amino acid resolution has been achieved (Hu et al., 2013). While initially limited to analysis of small proteins, recently HDX-MS has been used to study the dynamics of large protein complexes such as GroEL-nucleotide binding (Zhang et al., 2013) and antibody-antigen binding in viral capsids (Bereszczak et al., 2013).

Ion-mobility MS (IM-MS) allows one to separate coexisting forms of the same protein/protein complex that would otherwise be indistinguishable using MS alone. The time it takes an ion to traverse the ion mobility cell is related to its mass, charge, and rotationally averaged collision cross-section (CCS), the latter being a measure of the overall shape of the ion (Bohrer et al., 2008). Because of these capabilities, IM-MS has been used to study a number of challenging dynamic systems (Beveridge et al., 2013; Hilton et al., 2013) including aggregating proteins (Bernstein et al., 2009; Smith et al., 2010) and viruses (Uetrecht et al., 2011). Advances in computational tools to analyze the IM data have been limited; however, a program that aids such analysis has recently been developed (Sivalingam et al., 2013). Additionally, CCS measurements obtained from IM-MS experiments have been used as constraints to model protein complexes (Politis et al., 2010).

Thus far, only a few attempts have been made to use a combination of the above MS approaches in the context of protein modeling. In a recent example, a combination of XL-MS and HDX-MS was used to describe structural differences of NKR-P1A receptor (Rozbesky et al., 2012, 2013). Yet, it is still challenging for all the different MS approaches to be used for the study of a single system because each approach requires different sample preparation methods, mass spectrometers, and software setups for processing the raw data. Computational methods to make use of all different, but often complementary, MS information will also need to be developed.

### Detecting Conformational States of Assemblies in Cryo-ET Image Analysis

Cryo-EM has emerged as an important tool for studying structures of macromolecular complexes in different conformational states. Several successful applications performed 3D reconstructions of conformational states based on a reference-free classification of 2D images (Scheres et al., 2005; Spahn and Penczek, 2009; Elad et al., 2008). In such a procedure, the initial classification of particles is directly derived from the input data and does not rely on an initial knowledge of a template complex structure. Such reference-free methods are computationally significantly more challenging, but are essential for providing unbiased structural categorization of the complexes and their conformational states.

Recently, such methods have been extended to cryo-ET, opening new exciting opportunities for in situ analysis of complexes (Figure 1B). However, image analysis in cryo-ET is more challenging in comparison to single-particle reconstructions mainly due to higher noise levels (Frangakis and Förster, 2004) and lower nonisotropic resolution (Förster et al., 2005; Lucić et al., 2005). Among other issues, the “missing wedge effect” causes severe distortions in tomograms as a result of a maximal microscope tilt range from −70 to 70 degrees when collecting images. These missing data lead to anisotropic resolution and different kinds of artifacts that depend on structure and orientation of the object. In addition, difficulties arise in detecting and masking complexes in crowded and heterogeneous cellular tomograms (Beck et al., 2004; Brandt et al., 2010). Due to the higher noise level and lower resolution, it is crucial to classify and average a relatively large number of particles (Liu et al., 2012). However, the processing of 3D images is computationally more intensive. High-throughput classification of tens of thousands of subtomograms is not trivial and will demand novel, more efficient methods in several areas, including faster 3D alignment and classification methods together with automatic particle selection (Zhu et al., 2004), as well as the masking of target complexes from cellular tomograms (Xu and Alber, 2013).

Several types of reference-free subtomogram classification strategies have been adapted to cryo-ET analysis, including methods based on maximum likelihood approaches (Scheres et al., 2009) and methods that rely on iterative successive alignment and classification steps (i.e., the alignment-through-classification approach; Bartesaghi et al., 2008; Winkler, 2007; Winkler et al., 2009). In all these approaches, aligning the 3D subtomograms is one of the limiting factors in terms of computational efficiency. The alignment relies on the search for the rigid transformation of one subtomogram with respect to the second that maximizes the similarity measure between them. Several similarity measures take into account the missing wedge effects by using a constrained similarity score (Bartesaghi et al., 2008; Förster et al., 2008; Volkmann, 2010; Amat et al., 2010).

To find the optimal alignment, many existing methods use an exhaustive 3D scanning over all rotations/translations of one subtomogram relative to the second (Förster et al., 2008; Volkmann, 2010; Amat et al., 2010). Such methods are computationally intensive, which limits their applicability when dealing with large data sets. This problem becomes even more severe with increasing cryo-ET resolutions (Murata et al., 2010) and the resulting larger subtomogram volumes. Therefore, the development of new algorithms that improve speed and accuracy of high-throughput alignment is essential for increasing resolution and accuracy in structural characterization of complexes by cryo-ET. Recently, fast subtomogram alignment methods have been proposed to significantly speed up the alignment step. To enhance computational efficiency, they use a rapid computation of the best rotational transformation (Bartesaghi et al., 2008; Xu et al., 2012; Chen et al., 2013; Xu and Alber, 2013). These methods all rely in different ways on spherical harmonics-based fast rotational matching. They can enhance computational speed by more than three orders of magnitude in comparison to exhaustive scanning methods (Xu et al., 2012). Several other methods improved alignment accuracy by applying refinements given initial alignments (Bartesaghi et al., 2008; Xu and Alber, 2012; Kuybeda et al., 2013; Hrabe et al., 2012).

Fast and accurate subtomogram alignments are only one component for the successful classification of complexes in cryo-ET. Other challenges remain, which we cannot describe here in detail. Such challenges are for instance efficient and scalable approaches for dimension reduction (Heumann et al., 2011), which allow focusing on the information that is most relevant for discriminating the aligned subtomograms. Also methods are needed for automatic particle picking and masking from highly crowded cell tomograms (Xu and Alber, 2013), which is a prerequisite for generating the large amount of particles necessary for accurate classification and averaging of subtomograms to reconstruct density maps of assemblies in different states.

As an emerging and important imaging technique, the cost of 3D EM imaging is decreasing quickly. Combined with new detector technology and automation in data acquisition and analysis, it is becoming easier to generate large data sets of cryo-EM images or subtomograms of macromolecular complexes, which opens new opportunities to study large complexes at multiple conformations. Despite these advances (which in single-particle cryo-EM studies enabled the reach of near-atomic resolution; Hryc et al., 2011), in cryo-ET (Briggs, 2013) and most cryo-EM studies, the level of resolution that can be achieved is still far from allowing an atomic model to be directly constructed from the density.

### Pseudo-Atomic Models of Assemblies at Multiple Conformations Using EM Data

Generating a pseudo-atomic assembly model from most 3D EM density maps at intermediate to low resolution (∼5–25 Å) currently involves a series of steps, which often include segmentation (Pintilie et al., 2010) and fitting of multiple atomic models from either X-ray crystallography, NMR spectroscopy, structure simulation, or prediction methods (Esquivel-Rodríguez and Kihara, 2013; Degiacomi et al., 2013; Figures 1C and 1D). Identifying the optimal fit from a gamut of solutions is a challenging task, depending on the map resolution, the accuracy of the fitted model, the complexity of their representation, and the scoring function. Rigid fitting uses a global search in six translation/rotation degrees of freedom to get the best configuration of the atomic model in the map (Chacón and Wriggers, 2002; Volkmann and Hanein, 1999). For assembly modeling, multiple components have to be fitted, making it a multibody optimization problem that increases the search space exponentially with the number of components. So far, only a handful of assembly-fitting approaches have been developed that simultaneously optimize the position and orientation of the components within the 3D map (Kawabata, 2008; Lasker et al., 2009; Rusu and Birmanns, 2010; Zhang et al., 2010).

Because 3D EM maps can represent multiple conformational states, the conformation of the fitted components frequently has to be changed to gain insight into the dynamic properties of the assembly. Flexible fitting addresses this problem by improving the goodness-of-fit while simultaneously “flexing” the atomic structure in the map. Due to the complexity of the problem, this step is typically performed either by following the segmentation of the map after detecting the coarse assembly positions (Seitsonen et al., 2012) or in the context of the map using the internal symmetry (if the component approximate position is known and symmetry can be applied; Chan et al., 2011; Chapman et al., 2013).

Over the past decade, real-space refinement approaches have become very popular, using rigid-fragment fitting, low-parameter deformations, and fully flexible, all-atom gradient-descent and molecular dynamics optimizers (Chapman et al., 2013; Roseman, 2000; Schröder et al., 2007; Tama et al., 2004; Topf et al., 2008; Trabuco et al., 2008; DiMaio et al., 2009; Grubisic et al., 2010). These methods typically rely on a standard molecular mechanics force field with an additional biasing force calculated from the density map. They have been applied successfully, primarily at the intermediate resolution range, to a number of dynamic systems, including translocation intermediates in ribosomes (Agirrezabala et al., 2012) and ATP-triggered intermediates of the GroEL chaperonin (Clare et al., 2012). However, with the appropriate coarse-graining approach, they in principle can be applied to lower resolution maps, also coupled with hierarchical refinement at multiple stages to reduce overfitting (Pandurangan and Topf, 2012). Flexible fitting in combination with normal mode analysis (Tama et al., 2004; Suhre et al., 2006) has also been successful, mainly due to the ability to capture large-scale conformational changes, and is likely to play an important role in exploring the conformational states from multiple low-resolution maps. This approach helped for example in analyzing the dynamic toxic complex of anthrax at 18 Å resolution (Tama et al., 2006). An interesting recent development by Zhang and co-authors was to incorporate information from 2D classification of the electron micrographs into the refinement process without the need to include a 3D model of the density (Zhang et al., 2012a).

In sum, a wide selection of methods is available to model multiple conformations from intermediate- to low-resolution 3D EM maps. Mostly, the choice of the method depends on the size of the system under consideration, the level of flexibility, and other characteristics, such as the resolution and symmetry. The development of general flexible fitting methods robust enough to deal with different systems and with low-resolution maps remains a challenge. Moreover, progress in the development of new scoring functions (Vasishtan and Topf, 2011) and validation methods (Henderson et al., 2012) as well as in the integration of additional data from multiple sources (see below) will allow better characterization of the conformational states. Interestingly, a recent study demonstrated that the use of multiple flexible fitting approaches to achieve a consensus fit could aid in improving and qualitatively assessing such fits (Ahmed and Tama, 2013).

### Integrative Modeling

When refining assembly models in low-resolution density maps, the scoring function can also be expanded using spatial restraints generated from MS (Benesch and Ruotolo, 2011; Walzthoeni et al., 2013; Figure 1). Integration of data from multiple sources for assembly modeling has recently been implemented in a number of software packages (Loquet et al., 2012; Russel et al., 2012; Karaca and Bonvin, 2013). With XL-MS, data can be directly translated into distance restraints and combined with the 3D density fitting (as demonstrated by the structure determination of the 26S proteasome; Bohn et al., 2010; Lasker et al., 2012) or with 2D class-average images (Velázquez-Muriel et al., 2012). Systematic integration of XL-MS data with various complementary data types also assisted in the modeling of the bacterial signal recognition particle in complex with its receptor (Chu et al., 2004), and more recently, RNA polymerases (Chen et al., 2010; Blattner et al., 2011) and TRiC/CCT chaperonins (Herzog et al., 2012; Kalisman et al., 2012; Leitner et al., 2012a). HDX-MS, which can reveal relative solvent accessibility, has been used, for example, to study a multidomain kinase (Engen et al., 2013). Potentially, this method can probe the conformational dynamics of a wide range of other assemblies (Engen et al., 2013). Because the resolution of the method dictates the type of restraint that can be designed, which is clearly a nontrivial task, it is easier to use this information in the context of model assessment. Such an approach was taken recently to validate a pseudo-atomic model of the COPII cage assembly, which was flexibly fitted into an intermediate-resolution cryo-EM map (Noble et al., 2013). IM-MS data can be used to calculate CCSs and compared to the theoretical values calculated from models (Politis et al., 2010). A number of methods to calculate the theoretical CCS have been developed (Bleiholder et al., 2011; Mesleh et al., 1996; Shvartsburg and Jarrold, 1996). This information could be helpful, for example, in determining assembly component conformations (Hall et al., 2012).

With advances in the use of MS techniques to tackle heterogeneous samples of large complexes, including in situ (Zhang et al., 2009), it is likely to play an important role in modeling macromolecular assemblies in combination with 3D EM data, not only with respect to the overall architecture, but also to the conformational flexibility. However, many challenges in such data integration still remain. These include the difficulty in designing an accurate scoring function, the complexity of the conformational space to be sampled, the treatment of ambiguous input data, and model assessment. Moreover, it is necessary that all the experiments are performed at similar conditions to prevent differences in conformational dynamics between them. This may not always be the case. Future cryo-ET studies on specific tagged assemblies are likely to use XL-MS and other proteomics approaches to enable direct identification of specific assembly components captured in different conformations. Such methodology was recently proposed for identifying binding partners bound to ribosome dimers in situ (Ortiz et al., 2010).

### Summary

Cryo-EM/ET and MS can provide complementary information about macromolecular assembly structures in different conformational states. Computational methods have been an integral part of the acquisition and analysis of data originating from these techniques. In this Perspective, we have provided a brief description of the type of data obtained and the relevant computational methods used. We focused on XL-MS, HDX-MS, IM-MS, alignment, and classification methods of heterogeneous samples of cryo-ET subtomograms as well as the fitting of atomic models from various sources into 3D EM maps. We touched upon future developments that will push the boundaries of information gained from such approaches and will help capture the dynamics of assemblies. Finally, we discussed some of the challenges involved in the integration of this information. Such integration promises to increase our understanding of numerous biological processes.
